# Effect of Sodium Sulfite, Sodium Dodecyl Sulfate, and Urea on the Molecular Interactions and Properties of Whey Protein Isolate-Based Films

**DOI:** 10.3389/fchem.2016.00049

**Published:** 2017-01-18

**Authors:** Markus Schmid, Tobias K. Prinz, Andreas Stäbler, Sven Sängerlaub

**Affiliations:** ^1^Fraunhofer Institute for Process Engineering and Packaging IVVFreising, Germany; ^2^Chair for Food Packaging Technology, Technische Universität MünchenFreising, Germany

**Keywords:** whey protein, biopolymer, reactive additives, barrier properties, mechanical properties, protein solubility

## Abstract

Whey protein coatings and cast films are promising for use as food packaging materials. Ongoing research is endeavoring to reduce their permeability. The intention of this study was to evaluate the effect of the reactive additives sodium sulfite, sodium dodecyl sulfate (SDS), and urea on the oxygen barrier, water vapor barrier, and protein solubility of whey protein cast films. The concentration of the reactive additives was 1 to 20 wt.-%. Dried whey protein cast films were used as substrate materials. The water vapor transmission rate, the oxygen permeability, and the protein solubility were measured. Effective diffusion coefficients and effective sorption coefficients were calculated from the results of the water vapor sorption experiments. The presence of sodium sulfite resulted in an increased number of hydrophobic interactions and hydrogen bonds and a slightly decreased number of disulfide bonds. The oxygen permeability decreased from 68 to 46 cm^3^ (STP/standard temperature and pressure) 100 μm (m^2^ d bar)^−1^ for 1 wt.-% SDS in the whey protein cast film. The water vapor transmission rate decreased from 165 to 44 g 100 μm (m^2^ d)^−1^ measured at 50 to 0% r. h. for 20 wt.-% SDS in the whey protein cast film. The reduction in the water vapor transmission rate correlated with the lower effective diffusion coefficient.

## Introduction

Barrier packaging systems for foods and pharmaceuticals have to provide an oxygen barrier as well as a water vapor barrier in order to ensure adequate protection for the packed goods (Buchner, 1999). For this reason, multilayer plastic films containing one or more barrier layers are usually employed. A commonly used oxygen barrier material is the rather expensive ethylene vinyl alcohol copolymer (EVOH) (Lange and Wyser, 2003; Kucukpinar and Doruker, 2004; Müller, 2013). This petrochemical-based copolymer is neither renewable nor biodegradable (Schmid et al., 2012). An alternative is to use proteins, which have interesting properties that make them promising as a potential renewable substitute for EVOH in multilayer packaging (Cuq et al., 1998; Coltelli et al., 2016; Zink et al., 2016). Bugnicourt et al. (2013) and Schmid et al. (2012) showed that whey protein coatings and layers are of commercial relevance due to the fact that whey protein formulations have barrier properties of the same order of magnitude as synthetic barrier polymers. By including whey protein-based coatings in multilayer structures, this facilitates the recyclability of the multilayer films (Cinelli et al., 2016) and the biodegradability of specific multilayers can be maintained or even improved (Cinelli et al., 2014).

### Whey proteins

By technical definition, whey is the milk serum remaining from the production of cheese after removal of the curd. Whey has a dry matter content of 65 g per liter and a protein content of 6.0–7.0 g per liter (Sienkiewicz and Riedel, 1986; Kammerlehner, 2003). For the industrial production of whey protein it is necessary to extract this fraction from the liquid whey (Onwulata and Huth, 2008). β–lactoglobulin accounts for about half (53%) of all the whey protein in cows' milk and is thus its major component. It is a globular protein and its structure is stabilized by intramolecular interactions, such as disulfide bridges. Each monomer has two disulfide bridges and one free thiol group (Sienkiewicz, 1981a). The second most important whey protein is α–lactalbumin. This monomeric protein contains 123 amino acids, of which a high number is essential. α–lactalbumin possesses four disulfide bonds, but no free cysteine residues (Sienkiewicz, 1981b).

### Denaturation of proteins

Denaturation describes a reversible or irreversible change in the native conformation (secondary, tertiary, quaternary structure) without cleavage of covalent bonds, except for disulfide bridges. Therefore, denaturation can occur with any treatment that cleaves hydrogen bonds, ionic or hydrophobic bonds. This can take place due to a change in temperature, change in pH, effects of shear forces, addition of organic solvents, salts, urea, guanidine hydrochloride, or detergents, such as sodium dodecyl sulfate (Belitz et al., 2009). For film formation, protein denaturation followed by intermolecular interactions is required. Denaturation of globular proteins leads to unfolding of the molecule, thereby exposing reactive functional groups that are able to form new chemical bonds, such as disulfide bridges or physical linkages, such as van der Waals interactions, hydrogen bonding, and electrostatic and hydrophobic interactions (Onwulata and Huth, 2008).

### Intramolecular interactions

**Figure 1:**
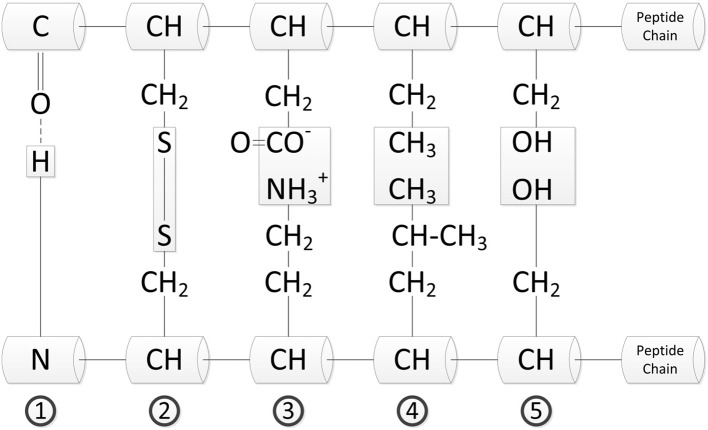
**Structure stabilization between peptide chains (Bannwarth et al., 2007)**.

Proteins exist in numerous spatial arrangements, which are stabilized by intermolecular and intramolecular interactions. The different types of interactions in proteins are shown in Figure 1. With a bond strength of −230 kJ/mol the covalent disulfide bridges (2) have the highest bond strength of the various interactions. They are followed by electrostatic interactions (3) having a bond strength of about −21 kJ/mol, which is the strongest non-covalent interaction. With a bond strength of about −15 kJ/mol the strength of hydrogen bonds (1) is only the third highest, but due to their high number in proteins they are of particular importance in protein folding. Although the hydrophobic interactions (4) of the nonpolar regions in the peptide chain play an important role for folding, the bond strength is about 0.1 kJ/mol per Å^2^ surface area and thus rather weak. Additionally, there are the dipole-dipole interactions (5). However, their binding force is lowest of all interactions in polypeptide chains (Belitz et al., 2009; Hammann and Schmid, 2014).

The aforementioned interactions determine the end properties of protein based films and coatings. Acquiring a better understanding of molecular interaction/property relationships is therefore not only of scientific relevance it is also important for the end industrial application. For example, these interactions affect the barrier performance of whey protein-based films significantly (Schmid et al., 2015). Furthermore, they affect the thermoformability of such films and coatings. It has been shown (Schmid, 2012; Stäbler and Schmid, 2016) that the post-crosslinking of whey protein-based coatings can lead to thermosetting behavior adversely affecting the thermoformability. Chemical modifications can be used to overcome these limitations (Zink et al., 2016).

### Reactive additives

Additives are able to reduce intermolecular interactions between the polypeptides, thus allowing the polymer chains to glide past each other during a subsequent thermoforming process. As these additives alter the secondary and tertiary structures of proteins by influencing the intramolecular interactions they function as denaturants (Brydson, 1999).

#### Sodium sulfite (Na_2_SO_3_) (sigma-aldrich chemie GmbH, steinheim, Germany)

The modification of protein chains with sodium sulfite (Na_2_SO_3_) is one option for reducing the chemical crosslinking. Na_2_SO_3_ inhibits free mercaptan groups and breaks down disulfide bonds (Thannhauser et al., 1984; Morel et al., 2000). In this redox reaction sulfite, as the reducing agent, transforms the disulfide bridges into two sulfonate groups (see Equation 1). It is a reversible process and the reverse reaction takes place in the presence of an oxidizing agent (Bailey and Cole, 1959).

(1) $$\text{R }-\text{ S }-\text{ S }-\text{ R }+\;{\text{SO}}_{3}^{2-}\;⇋\text{ RS }-\;{\text{SO}}_{3}^{2-}\;+\;{\text{RS}}^{-}$$

#### Sodium dodecyl sulfate (SDS) (sigma-aldrich chemie GmbH, steinheim, Germany)

In the presence of sodium dodecyl sulfate (SDS) proteins form SDS-protein complexes that are negatively charged (Jung et al., 2008). As a result, the non-covalent interactions between the protein chains are cleaved. Therefore, SDS breaks the hydrophobic interactions and hydrogen bonds, while the disulfide bridges stay intact. SDS is a detergent with a long hydrophobic tail and an anionic and hydrophilic head and is also used as a surfactant (Liu and Hsieh, 2008; Rehm and Letzel, 2010; Vančik, 2014).

#### Urea (merck KGaA, darmstadt, Germany)

Similar to SDS, urea is known to break non-covalent interactions. It is more efficient at breaking hydrogen bonds than hydrophobic interactions (Zhou et al., 2008; Manoi and Rizvi, 2009). Urea, as a polar non-electrolyte, is able to form hydrogen bonds with the peptide backbone and charged species as well as with water molecules present in the solution. The ability of a urea molecule to form hydrogen bonds is twice as high as a water molecule (O'Brien et al., 2007; Liu and Hsieh, 2008).

### Aim of the study

The aim of this study was to evaluate the effect of the reactive additives, such as sodium sulfite (Na_2_SO_3_), sodium dodecyl sulfate (SDS), and urea on molecular interactions and the resulting oxygen and water vapor permeability and mechanical properties of whey protein-based cast films.

## Materials and methods

### Film preparation

#### Preparation of the solution

To achieve a homogeneous solution, 10% (w/w) whey protein isolate (BiPRO®, Davisco Foods International (Le Sueur, Minnesota, MN, USA)) was added to distilled water and stirred for 30 min at 200 rpm in an electric stirrer [Thermomix 31-1, Vorwerk Elektrowerk GmbH & Co KG (Wuppertal, Germany)]. Afterwards, the additives were added to the rotating Thermomix and the solution was heated to 90°C and stirred for another 30 min at 200 rpm to denature the protein. As plasticizer, 66.7% (w/w protein) glycerol was added, with the percentage here referring to the actual amount of protein in the solution. Glycerol is a commonly used plasticizer for biopolymer films (Gao et al., 2006; Aritonang and Melia, 2014; Jost et al., 2014; Jost and Langowski, 2015; Boy et al., 2016; Jost and Stramm, 2016). To avoid gel formation and to mix the components, the stirrer temperature was kept at 50°C for an additional 30 min at 200 rpm. In order to remove small particles, the liquid was filtered with an ordinary French press. Samples having a high viscosity and a high number of air bubbles were placed in an ultrasonic bath at 50°C and treated at 37 kHz for 20 min. The actual weights used in the different formulations are presented in Table 1. Suitable concentrations of the active additives (sodium sulfite, urea, SDS) were determined empirically. Higher concentrations caused gelling and prevented further processing.

**Table 1:** **Compositions by weight of the 750 g cast formulations**.

**Sample**	**WPI/g**	**Distilled water/g**	**Plasticizer/g**	**Added additives/g**		
				**Na_2_SO_3_**	**Urea**	**SDS**
Reference	78.9	621.0	50.0	—	—	—
1 wt.-% Na_2_SO_3_	78.9	620.3	50.0	0.8	—	—
2 wt.-% Na_2_SO_3_	78.9	619.5	50.0	1.5	—	—
3 wt.-% Na_2_SO_3_	78.9	618.8	50.0	2.3	—	—
5 wt.-% urea	78.9	617.3	50.0	—	3.8	—
10 wt.-% urea	78.9	613.5	50.0	—	7.5	—
13.5 wt.-% urea	78.9	610.9	50.0	—	10.1	—
1 wt.-% SDS	78.9	620.3	50.0	—	—	0.8
10 wt.-% SDS	78.9	613.5	50.0	—	—	7.5
20 wt.-% SDS	78.9	606.0	50.0	—	—	15

#### Film casting and drying

A consistent film thickness plays an important role for the curing process. Therefore, the amount of solution per Petri dish was calculated using the following equation (Equation 2).

(2) $$\begin{array}{l} \text{ solution/g}=\\ \frac{\text{area petri dish}/{\text{cm}}^{2}\;\cdot \text{ film thickness}/\text{cm }\cdot \text{ mass g }\cdot \text{ density}/\frac{\text{g}}{{\text{cm}}^{3}}}{\text{dry matter}/\text{g}} \end{array}$$

The density of the solution was 1.4 g/cm^3^. The value was taken from other studies of our group (Schmid et al., 2014). The calculated amount of solution for a film thickness of 200 μm was filled into the Petri dishes with a syringe. After that the air bubbles were removed to get an even surface. The finished cast films were cured in a conditioning chamber at 23°C and 50% r.h. for at least 9 days on a leveled shelf to avoid uneven film thicknesses.

### Applied theory for barrier and permeation

To describe the diffusion and permeation properties the equations of Fick were applied (Fick, 1855; Foth, 2005). The model is similar to the solution-diffusion model of Graham (Graham, 1866a,b). The theory and equations of permeation and diffusion according to Fick's equations and the model are described elsewhere (Fick, 1855; Barrer, 1941; Crank, 1975; Koros, 1990; Gavara et al., 1996; Langowski, 2008; Krevelen and Nijenhuis, 2009; Miesbauer and Langowski, 2010).

The permeability coefficient P is the product of the sorption coefficient S and the diffusion coefficient D (Equation 3) (Koros, 1990; Gavara et al., 1996; Krevelen and Nijenhuis, 2009).

(3) P = S · D

The boundary conditions for the validity of the applied model are: (1) The sorption behavior can be described by Henry's law, i.e., the sorption coefficient S is a constant (Henry, 1803; Crank, 1975; Krevelen and Nijenhuis, 2009). (2) The permeation process can be described by Fick's laws. (3) The diffusion coefficient is constant. (4) The diffusion in the polymer matrix has a higher rate than the sorption at the surface (Crank, 1975; Marais et al., 2000).

Whey protein has polar groups and interacts with water vapor (Berlin et al., 1968, 1973; Coupland et al., 2000). Therefore, strictly speaking, Henry's law is not valid for the interaction with water vapor. Nonetheless, the equations can be applied but the results are approximations. To emphasize that these results are approximations, the results of the water vapor sorption experiments are attributed with the term “effective” (Schmid et al., 2014).

### Measurement methods

#### Film thickness

To determine the film thickness a Precision Thickness Gauge (Model FT3 by Rhopoint Instruments, Bexhill on Sea, UK) was used. For accurate measurement, five different positions on every specimen were measured. The measured film thicknesses were in the range of 162–258 μm. The respective thickness of each characterized specimen was considered for calculation of the results.

#### Protein solubility study

As a first step the stored samples were removed from the Petri dishes and cut into small pieces. Subsequently they were frozen with liquid nitrogen and ground using a laboratory mill (ZM100, F. Kurt Retsch GmbH & Co. KG, Haan, Germany) equipped with a 0.5 mm sieve to obtain a homogenous powder. After the milling, the powder was stored in a sample tube at 1°C until use. The extraction of the samples with different buffer systems as well as the determination of the protein solubility are described elsewhere (Hammann and Schmid, 2014; Schmid et al., 2015). All measurements were carried out in duplicate.

#### Mechanical properties

To determine the mechanical properties, the cast films were analyzed using a heatable (Type: TEE 52/RX, Brabender Realtest GmbH, Moers, Germany) and a non-heatable (Z005, Zwick GmbH & Co. KG, Ulm, Germany) tensile testing machine. First of all the samples were sliced to a width of 15 mm and the clamping length of 50 mm was marked. For accurate measurement the thickness of every stripe was also determined with the Precision Thickness Gauge FT3 (Hanatek, St. Leonards-on-Sea, UK).

The tensile tests on the heatable machine were performed at 100°C, whereby the elongation at break and tensile strength were measured. Furthermore additional measurements were performed on the non-heatable tensile testing machine at 23°C in order to determine the elongation at break, tensile strength, and Young's modulus.

As such all the mechanical properties were determined in accordance with the DIN EN ISO 527 (DIN, 2012) standard.

#### Oxygen permeability

The oxygen permeability was determined with an OxTran Twin (MOCON Inc., Minneapolis, USA) in accordance with DIN 53380-3 in an air-conditioned laboratory at 23°C and 50% r.h. (DIN, 1998). For the reference and the samples with Na_2_SO_3_ three specimens were measured, while for the other films only two specimens were analyzed. The condition for defining the end of the measurement was attainment of a steady state for at least 10 h. To obtain the Q_100_-values the results were normalized to a thickness of 100 μm, by dividing the results by the corresponding thickness and then by multiplying by 100.

#### Water vapor transmission rate (WVTR)

The WVTR was measured by the gravimetric method, according to DIN 53122-1. For every film four specimens were measured at a temperature of 23°C and a relative humidity of 50%. As a high water vapor transmission rate was expected, the samples were masked. This means they were laminated with a high barrier adhesive aluminum foil from both sides. This foil had a round opening of area 10 cm^2^, which represented the measuring area. The remaining experimental setup as well as the calculation of the WVTR was performed according to Schmid et al. (2014).

#### Water vapor sorption

First the cast films were cut into small slices with an area of a few square centimeters and thickness measurements were performed. To remove the water completely, the films were stored in a desiccator filled with silica gel for 1 week and the dry weight was determined. For measurement the samples were stored in a conditioning chamber at 23°C and 50% r.h. To ascertain the water vapor absorption, the cast films were weighed at certain intervals, first every 2 min then every 10 min, until an equilibrium with the ambient conditions had been established. For every formulation three samples were measured.

##### Determination of the effective sorption coefficient S_eff_

The ratio of the water vapor absorption to the dry weight of the film was determined using Equation (4).

(4) $${\text{C}}_{{\text{H}}_{2}\text{O},\text{t }=\;\infty }=\frac{{\text{m}}_{{\text{H}}_{2}\text{O},\text{t }=\;\infty }}{{\text{m}}_{\text{dry film}}}$$

The effective sorption coefficient was calculated from the water vapor absorption of the sample, the water vapor pressure at 23°C and 50% r.h. (1404 Pa), and the density of the whey protein film (1.4 g/cm^3^) according to Equation 5 (Lück, 1992). The density change on water vapor absorption was neglected. The unit of *S*_*eff*_ is cm^3^ (STP) (cm^3^ Pa)^−1^. STP stands for standard pressure and temperature. Water vapor is treated as an ideal gas, i.e., at STP one mole of water has a volume of 22414 cm^3^ STP (molar volume) (Foth, 2006).

(5) $$S_{eff\cdot }=\frac{{\text{C}}_{{\text{H}}_{2}\text{O},\text{t }=\;\infty }}{{\text{p}}_{{\text{H}}_{2}\text{O}}}$$

**Figure 2:**
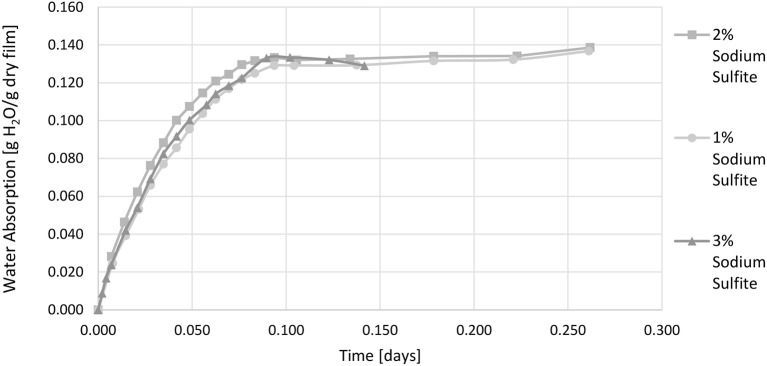
**Water vapor absorption of films with 1 wt.-%, 2 wt.-%, and 3 wt.-% sodium sulfite 23°C and 50% r.h. (relative humidity)**.

As the sorption coefficient of whey is dependent on the relative humidity it is called the effective sorption coefficient in this study. It is valid only for the temperature and relative humidity applied during testing. Figure 2 shows the water vapor absorption at 50% r.h. as a function of time. From the water vapor absorption at equilibrium the effective sorption coefficient was calculated.

##### Determination of the effective diffusion coefficient D_eff_

**Figure 3:**
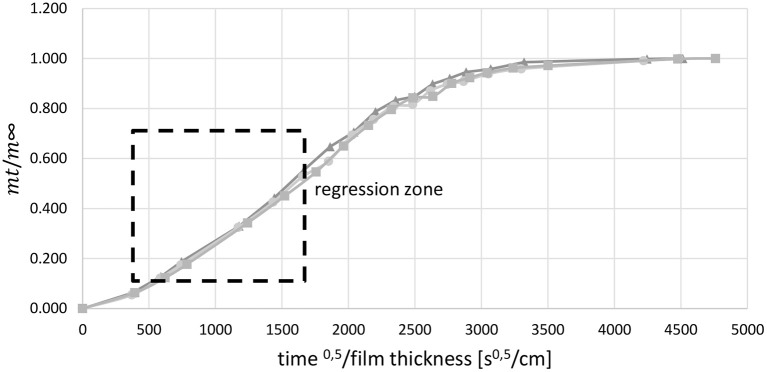
**Regression zone for the determination of *D*_*eff*_, 3-fold determination, example for film with 1 wt.-% SDS**.

In order to identify the effective diffusion coefficient the relative saturation was plotted as a function of the square root of time divided by the film thickness. The relative saturation is the ratio of the weight of water in the film at time t (m_t_) and the weight of water in the film at saturation (m_∞_). Between a relative saturation of 0.1 and 0.7 the graph is almost a straight line. Within this regression zone (Figure 3) a line of best fit was drawn through the reference points. The slope of this line was used to determine the effective diffusion coefficient by using Crank's law (Equation 6, Crank, 1975).

(6) $$\frac{{\text{m}}_{{\text{H}}_{2}\text{O},\text{time}}}{{\text{m}}_{{\text{H}}_{2}\text{O},\text{time }=\;\infty }}\;=\;\frac{4}{\sqrt{\pi }}\cdot \sqrt{\frac{D_{eff\cdot }\cdot \text{time}}{{\text{thickness}}^{2}}}$$

##### Calculation of the effective permeation coefficient P_eff_

The effective permeation coefficient is the product of the effective sorption coefficient and the effective diffusion coefficient. The values are standardized to a thickness of 100 μm. In this case the value is an effective water vapor permeability. The standardization is reached by dividing the measured value by the actual thickness of the film and multiplying the result by 100 μm.

#### Statistical methods

For all the experiments a completely randomized experimental design was used and statistics were applied as described elsewhere (Schmid et al., 2013). Therefore, the run sequence of the experimental units was determined randomly. Randomization was performed by the computer program Visual-XSel 12.0 Mulivar (CRGRAPH, Munich, Germany). The Kolmogorov-Smirnov-Test procedure in Visual-XSel 12.0 Multivar with a level of significance defined at 5% was applied in order to investigate if a normal distribution adequately describes the sets of data which were obtained (in the case of measurements with at least threefold determination). The hypothesis of normality was validated in all cases. Therefore, calculated standard deviations are given as error bars in the figures. An exception to this is the oxygen permeability measurements which was performed with twofold determination and therefore the minimum and maximum values are given as error bars.

## Results and discussion

### Protein solubility study

The protein solubility studies showed an effect of the reactive additives on the intermolecular interactions of protein chains. The results are presented in Table 2 (Decision matrix of intermolecular interactions), which shows the properties of all the samples normalized to the reference. Thereby a “0” means that the sample was at almost the same level as the reference, while a “+” or “−” means a slightly increased or decreased rate. Furthermore, the double usage of these (“++”;“−−”) implements a major change. Regarding the addition of Na_2_SO_3_ to the film forming solution it is noticeable that the relevance of both hydrophobic interactions and hydrogen bonds shows a strong increase, while the contribution of disulfide bonds shows a slight decrease for all concentrations.

**Table 2:** **Decision matrix of intermolecular interactions (“0” means that the sample was at almost the same level as the reference, while a “+” or “−” means a slightly increased or decreased rate**.

	**Na**_**2**_**SO**_**3**_			**SDS**				**Urea**	
	**1 wt.-%**	**2 wt.-%**	**3 wt.-%**	**1 wt.-%**	**10 wt.-%**	**20 wt.-%**	**5 wt.-%**	**10 wt.-%**	**13.5 wt.-%**
Hydrophobic Interactions	++	++	++	0	0	0	0	0	0
Hydrogen Bonds	++	++	++	0	−	−	0	0	0
Disulfide bonds	−	−	−	0	+	+	0	0	0

*The double usage of these (“++”;”−−") implements a major change*.

In contrast, the SDS films show an effect at higher concentrations and only for the relevance of hydrogen and disulfide bonds. While the importance of hydrogen bonds is slightly decreased, the contribution of disulfide bonds is slightly increased. The addition of urea did not show any significant effects. As explained in the introduction, it was expected that SDS would also decrease hydrophobic interactions. However, the results from this protein solubility study did not show such behavior.

### Mechanical properties

#### Elongation at break

The following subsections describe the elongation at break at 23° and 100°C.

##### Elongation at break measured at 23°C

**Figure 4:**
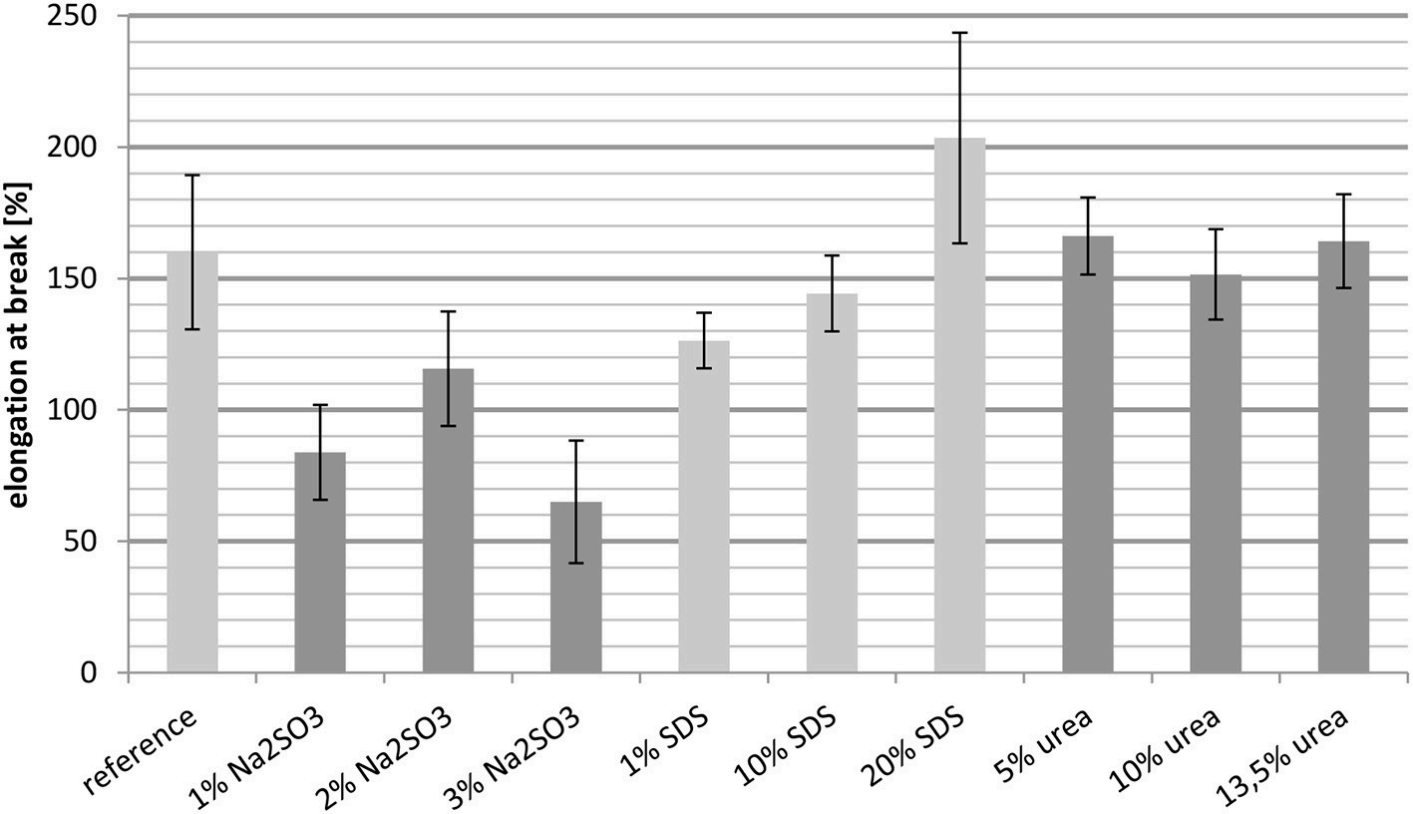
**Elongation at break measured at 23°C**.

The results in Figure 4 show the elongation at break of the untreated whey protein cast film and films containing different concentrations of Na_2_SO_3_, SDS, and urea. In order to obtain the desired property of the film, i.e., good thermoformability, a high elongation at break is desired.

Compared to the reference, the elongation at break was reduced for the three samples containing Na_2_SO_3_. Even an increased concentration did not result in the desired effect, although the elongation at break was slightly higher with 2 wt.-% Na_2_SO_3_ compared to 1 wt.-% and 3 wt.-%.

In contrast, the addition of SDS resulted in a nearly linear increase in the elongation at break. The films containing 1 wt.-% and 10 wt.-% SDS had a value lower than the reference, whereas the film with 20 wt.-% SDS showed a significant increase. Thus the whey protein film with 20% SDS addition had the best performance.

For the cast films containing urea, it is noticeable that the elongation at break was almost constant for the different concentrations. Furthermore, these films had the same elongation at break as the reference. Therefore, urea was not able to improve the thermoformability of the films.

Overall, Na_2_SO_3_ increases the relative importance of hydrogen bonds (Table 2) and they play the biggest role in the crosslinking of whey films. A reduced elongation at break is an indication of denser and less flexible crosslinking. SDS increases the role of disulfide bridges, which have high bond strength. The increased elongation at break can be explained by the ability of SDS to break hydrophobic interactions and hydrogen bonds, which causes less intermolecular interactions which might not have been compensated by additional disulfide bridges.

##### Elongation at break measured at 100°C

**Figure 5:**
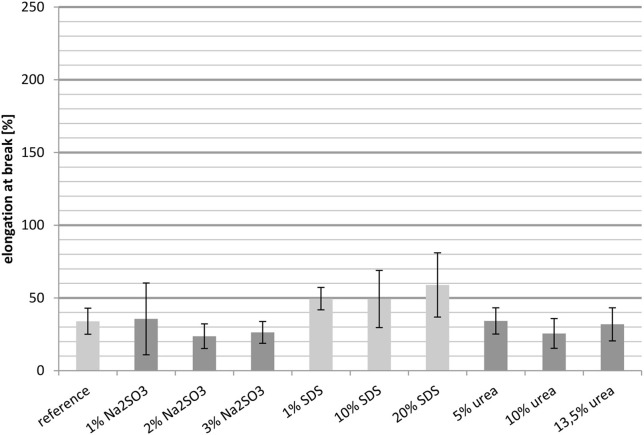
**Elongation at break measured at 100°C**.

The elongation at break of whey protein based cast films decreased with increasing temperature. Comparison of the elongation at break at 23°C (Figure 4) with the values determined at 100°C (Figure 5) it is clear that the elongation at break at 100°C, regardless of the added substance, cannot attain 100%. In contrast the elongation at break at 23°C ranged from 65% (3 wt.-% Na_2_SO_3_) to approximately 200% (20 wt.-% SDS). The decreased elongation at break at 100°C was probably caused by the thermally introduced breaking of disulfide bridges.

It is noteworthy that the samples, relative to the reference, showed better results than at 23°C, which might have resulted from the disproportionately high decrease in elongation at break in the reference with increasing temperature. At 100°C only the samples containing Na_2_SO_3_ as additive showed almost the same level as the reference. However, it was not possible to distinguish between the different concentrations due to the high standard deviation. Although, the films containing 1 wt.-% and 10 wt.-% SDS showed almost the same level of elongation at break, the value obtained with 20 wt.-% SDS was tendentially slightly higher and thus the highest of all the films. However, due to the generally high standard deviations a clear conclusion cannot be drawn from the results.

#### Tensile strength

##### Tensile strength at 23°C

Besides the elongation at break, the tensile strength was also measured at 23° and 100°C. In general the tensile strength behaves the opposite toward crosslinking as the elongation. An increase in the degree of crosslinking raises the value of the tensile strength.

**Figure 6:**
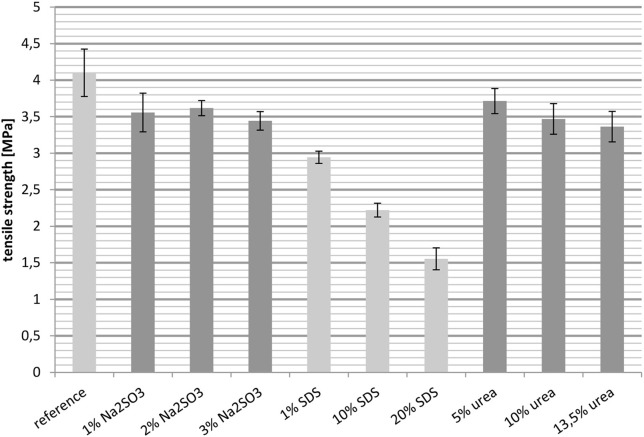
**Tensile strength measured at 23°C**.

Figure 6 shows the tensile strength of the films at 23°C, with the reference having the highest value. Both the cast films treated with Na_2_SO_3_ and those treated with urea gave similar results, although the intermolecular interactions were quite different. However, while an increasing urea content resulted in a slightly downward trend, an increase in the Na_2_SO_3_ concentration had no effect. The tensile strengths of the films containing SDS were the lowest, decreasing from 3 MPa for 1 wt.-% SDS to 1.5 MPa for 20 wt.-% SDS.

All in all it was unexpected that SDS reduces the tensile strength even though it increases the importance of disulfide briges. An explanation for this may lie in the ability of SDS to break hydrophobic interactions and hydrogen bonds. Additionally, the concentration of SDS was high, which can further reduce the tensile strength. In contrast, the increasing relevance of hydrogen bonds as well as hydrophobic interactions had almost no significant effect on the tensile strength.

##### Tensile strength at 100°C

**Figure 7:**
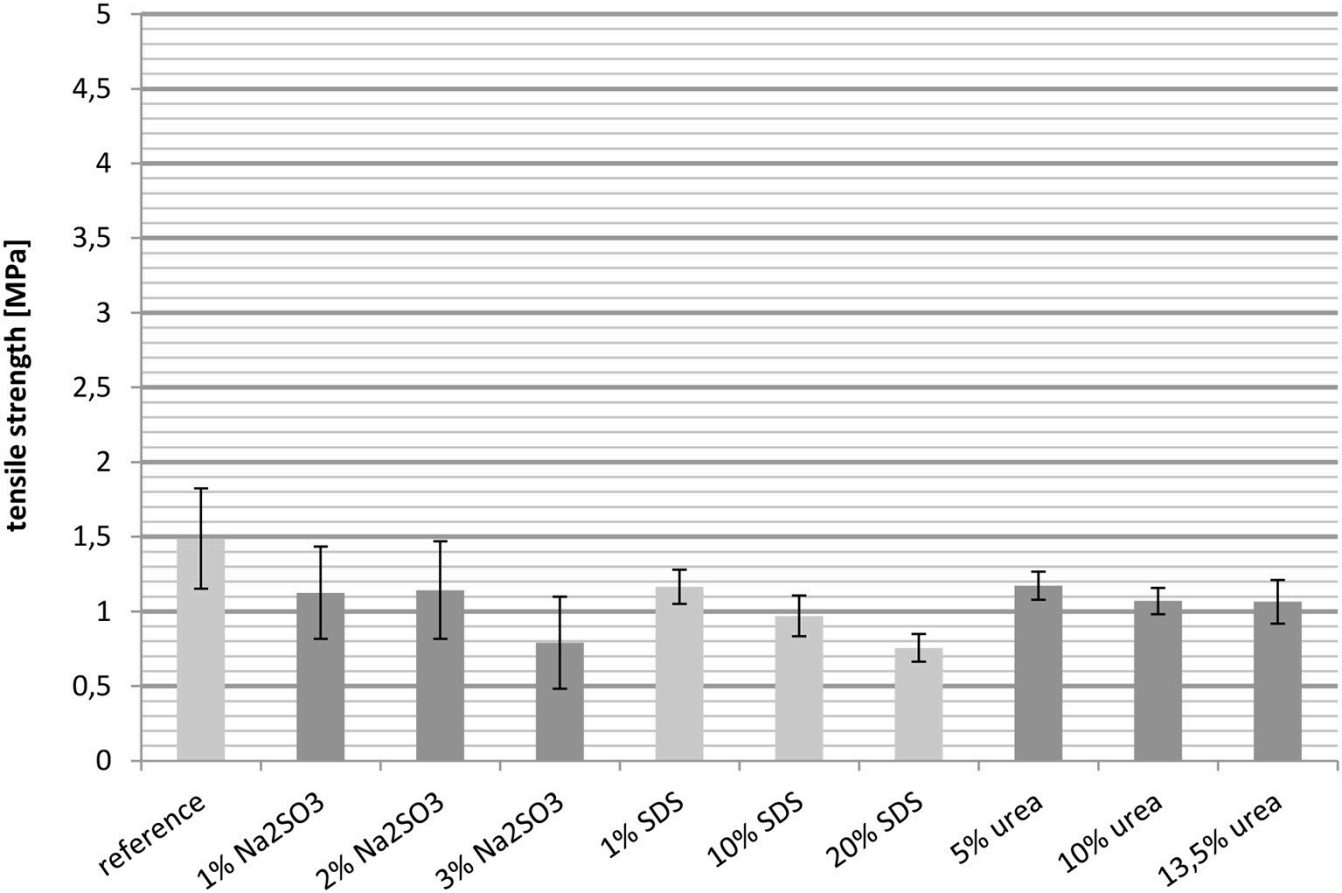
**Tensile strength measured at 100°C**.

Just like the elongation at break, the tensile strength decreased when the measurement temperature was increased from 23° to 100°C. The tensile strength of the reference at 100°C was only one third of the value determined at 23°C. None of the films (Figure 7) exceeded the tensile strength of the reference and the values determined at 100°C were 50 to 80% lower than the values determined at 23°C.

In this study it was observed for whey protein based cast films that a high tensile strength goes hand in hand with a low elongation at break and vice versa. Interestingly, it was observed that an increasing the importance of disulfide bridges in the network still causes a decreasing tensile strength. This can be explained by the reduced number of hydrophobic interactions and hydrogen bonds and the weakening of the network due to the presence of SDS. So overall it is rather difficult to correlate the tensile strength with the intermolecular interactions in the films.

#### Young's modulus

**Figure 8:**
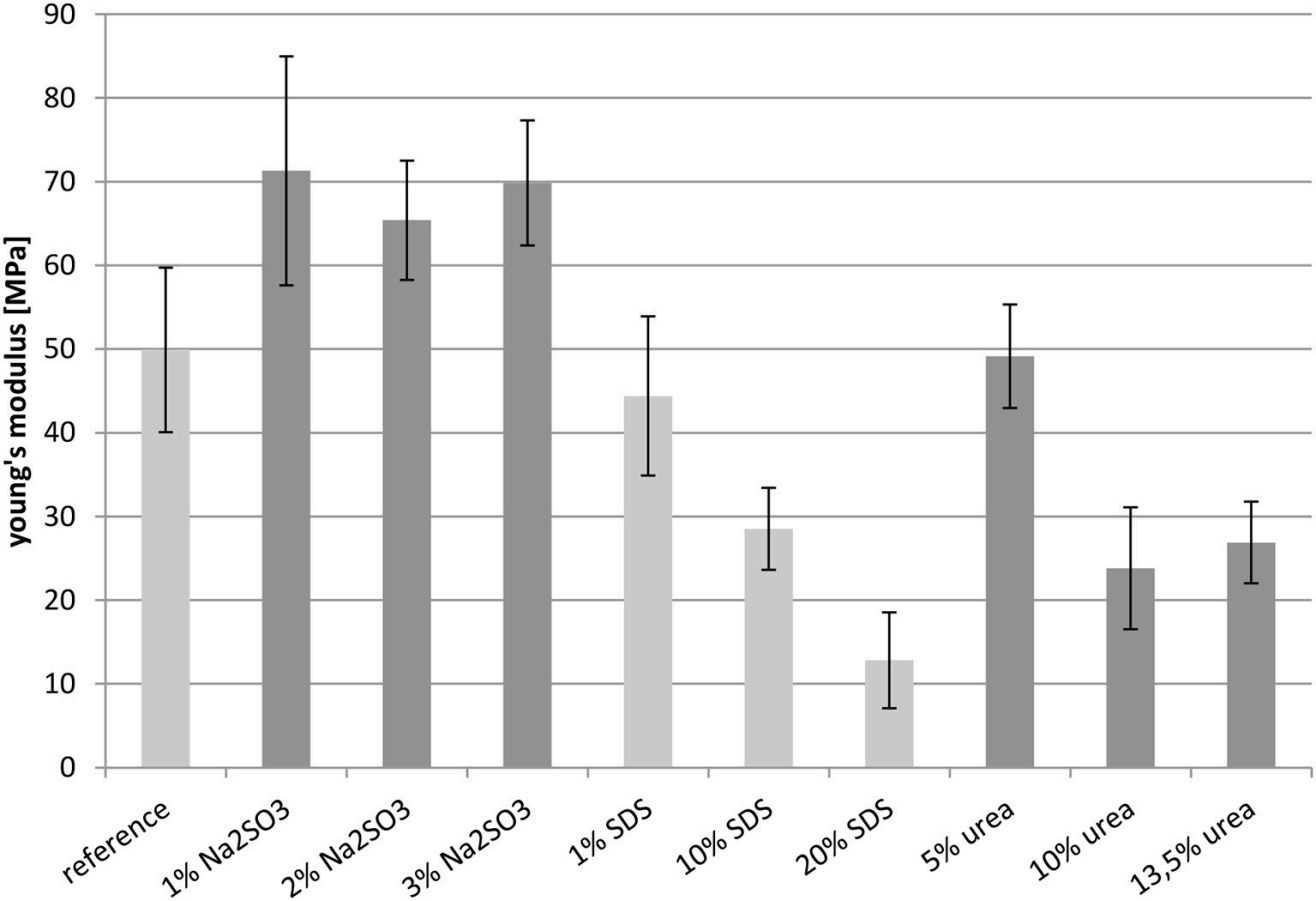
**Young's modulus measured at 23°C**.

Figure 8 shows the Young's moduli of the films. The samples containing Na_2_SO_3_ showed quite similar Young's moduli, and these values were higher than the reference. The value of the reference and the sample treated with 1 wt.-% SDS were almost equal. However, the Young's moduli of the films containing SDS decreased from 44 MPa to about 10 MPa with increasing additive content. The film treated with the lowest urea concentration of 5 wt.-%, had a Young's modulus of 50 MPa, which corresponded to the reference. This value was halved by doubling the urea concentration, with an additional increase having almost no effect.

All in all the results concerning the Young's modulus were rather surprising. It was expected that films with a high contribution of disulfide bridges would be rather stiff and thus have a high Young's modulus, whereas the films only stabilized by weaker non-covalent forces would be more elastic as the peptide chains would glide along each other more easily. However, this expectation was not confirmed by these measurements. On the contrary, films with no covalent interactions (sodium sulfite as additive) showed a high Young's modulus, whereas the lowest Young's moduli were observed for the protein films only stabilized by disulfide bridges (high content of SDS or urea). This effect might be explained by electrostatic interactions introduced into the network by the SDS and sodium sulfite. This kind of interaction cannot be determined by the buffer systems used here and further investigations are necessary.

#### Summary of the mechanical tests

The majority of the measurements showed that an increased elongation at break was accompanied by decreased tensile strength and a decreased Young's modulus. For example, raising the SDS concentration led to an increased elongation at break, while the tensile strength and the Young's modulus decreased. Comparing the samples with the reference, it is notable that the films treated with Na_2_SO_3_ could not match the reference in most cases. Also, the cast films with urea fell short of expectations. Their mechanical properties were in most cases similar to those of the reference. The exceptions were the samples with higher concentrations of urea which showed slightly decreased Young's moduli. The films containing 1 wt.-% SDS did not have improved mechanical properties compared to the reference. However, a significant effect was observed when SDS was applied in high concentration. It was also observed that the mechanical properties deteriorated drastically when the temperature was increased to 100°C. The elongation at break decreased with rising temperature, as did the tensile strength. However, comparing the values with the reference, the relative improvement in performance achieved by the additives was higher than at 23°C. For example, the reference at 23°C had an elongation at break of 150% and the elongation at break of the best sample, with 20 wt.-% SDS, was improved to 200%, while the reference at 100°C only reached about 35%, yet the best film with nearly 60% was almost twice as high. These measurements show that treatment with certain additives provides a suitable method for influencing the mechanical properties of protein based films.

The most important mechanical property regarding thermoformability is probably the elongation at break. As the films are stretched during the thermoforming process, an elongation at break as high as possible is desirable in order to prevent tearing. Below, the elongation at break is compared to the results of the protein solubility study.

It is notable that all films with Na_2_SO_3_ had a very poor or poor elongation at break compared to the reference. The intermolecular interactions in those films consisted primarily of hydrophobic interactions and hydrogen bonds. Despite this, Na_2_SO_3_ had a negative effect on the elongation at break of the cast films and was therefore not a suitable additive for improving the thermoformability. Furthermore, the addition of urea seemed to have no effect at all. Neither the intermolecular interactions nor the elongation at break increased on adding urea. The most promising additive was SDS, although the elongation at break of the films with 1 and 10 wt.-% SDS was lower than that of the reference. The film with 20 wt.-% SDS showed an increased elongation at break. This can be explained by the increased relevance of disulfide bridges. However, the hydrophobic interactions and hydrogen bonds should not be disregarded, while the elongation at break increased, also the hydrophobic interactions and the hydrogen bonds slightly raised. This indicates that a slightly increased level of hydrophobic interactions, combined with fewer hydrogen bonds and as many disulfide bridges as possible, may lead to a better elongation at break. Overall it seems that strong covalent interactions, such as disulfide bridges are necessary to stabilize the protein network and therefore improve the elongation at break. Accordingly, a low number of non-covalent interactions is another important factor because this generally causes enhanced molecular movability.

### Oxygen permeability

**Figure 9:**
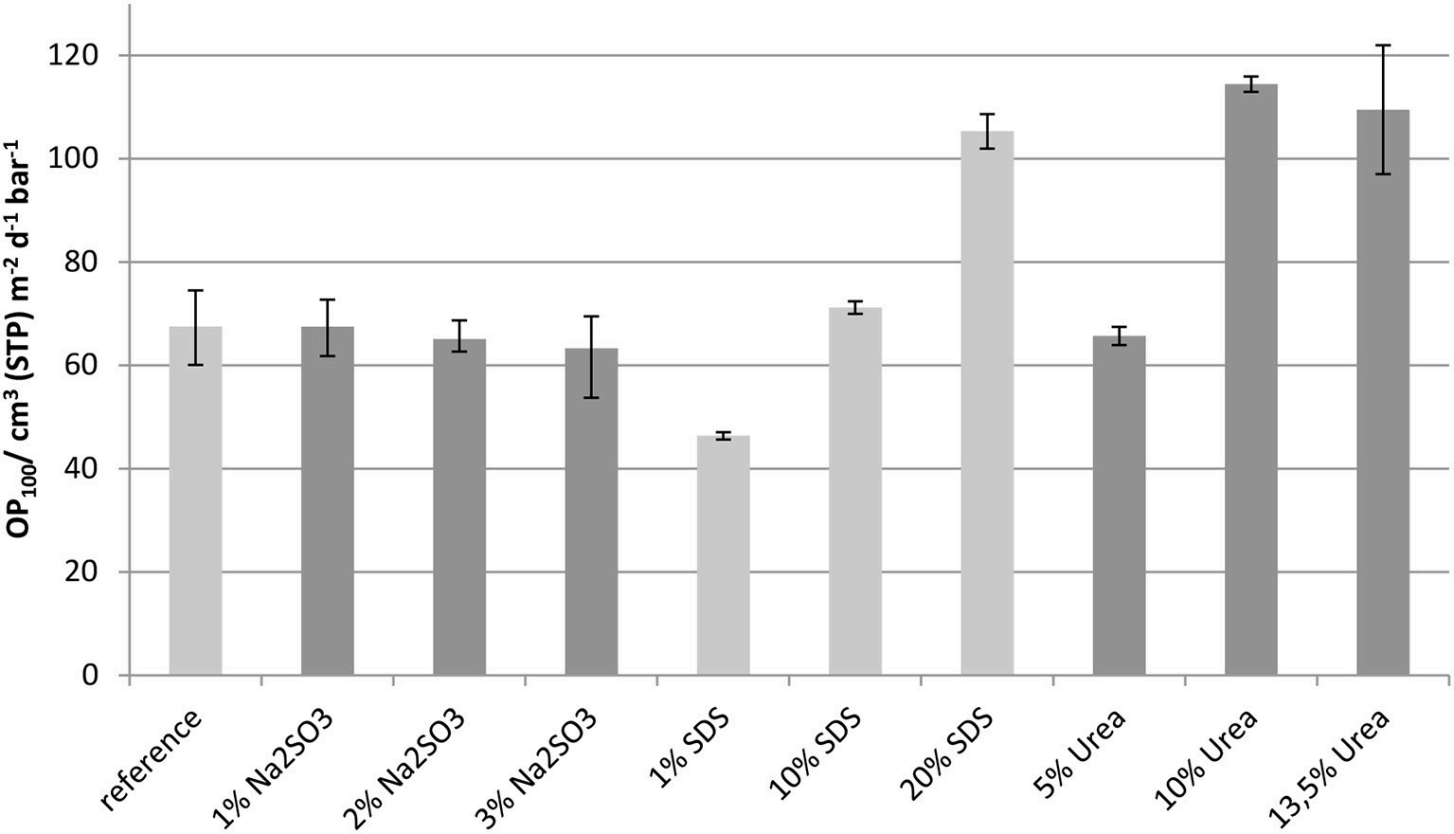
**Oxygen permeability (OP_100_) measured at 23°C and 50% r.h**.

The results of the oxygen permeability measurements, which were normalized to a film thickness of 100 μm, are presented in Figure 9. The error bars show the maximum and minimum values measured.

The addition of Na_2_SO_3_ in concentrations up to 3 wt.-% had no significant effect on the oxygen permeability. As a further increase in Na_2_SO_3_-content resulted in formulation viscosities too high for film casting, it was not possible to extend the studies to higher concentrations. As the solubility studies showed that disulfide bonds were cleaved by the addition of Na_2_SO_3_ (Table 2) it can be assumed that disulfide bonds have little impact on the oxygen permeability. In contrast, a low concentration of SDS slightly reduced the oxygen permeability of whey protein based films. However, with increasing SDS content in the films the oxygen permeability increased gradually, even above that of the reference. Such behavior can be explained by better crosslinking, such as by disulfide bridges, which is compromised at higher concentration due to disturbance of the protein network. Even though 1 wt.-% SDS showed no measurable effect on disulfide bridges (Table 2) it can be assumed that even a low concentration led to better crosslinking. The addition of urea, which above all splits hydrogen bonds, increased the oxygen permeability as expected because hydrogen bonds are of high importance for reducing the oxygen permeability of whey protein based films and coatings (Schmid et al., 2015). Films with concentrations above 10 wt.-% do not show a further significant change in oxygen permeability.

### Water vapor transmission rate

The water vapor transmission rate data are plotted in Figure 11. The addition of Na_2_SO_3_ as well as the addition of SDS in concentrations up to 10 wt.-% had no significant effect on the WVTR of whey protein based films. However, the addition of 20 wt.-% SDS significantly reduced the WVTR of the films. These films are particularly noteworthy as the WVTR was reduced to 44 g m^−2^ d^−1^ and thus to less than one third of the reference value. A hypothesis for explaining the low WVTR is the combination of three effects: (1) SDS reduces the number of hydrogen bonds and this could therefore result in a structure that swells less or interacts less with water molecules. (2) SDS has a relatively long hydrophobic tail which behaves water repellant. Also, the hydrophobicity of the tail would readily explain the higher oxygen permeability for 20 wt.-% SDS. The polar SO_4_-‘headgroup’ is likely to be bound to active groups of whey protein and therefore would be less prone to react with water. (3) SDS increases the importance of disulfide bridges as observed in the protein solubility study, which leads to a decrease in the free volume (Table 2). In contrast, the addition of urea led to an increase in the water vapor transmission rate, whereby the higher the urea concentration the higher the WVTR. As urea readily splits hydrogen bonds it is possible that these are occupied by water molecules, therefore increasing the WVTR.

### Water vapor sorption

**Figure 10:**
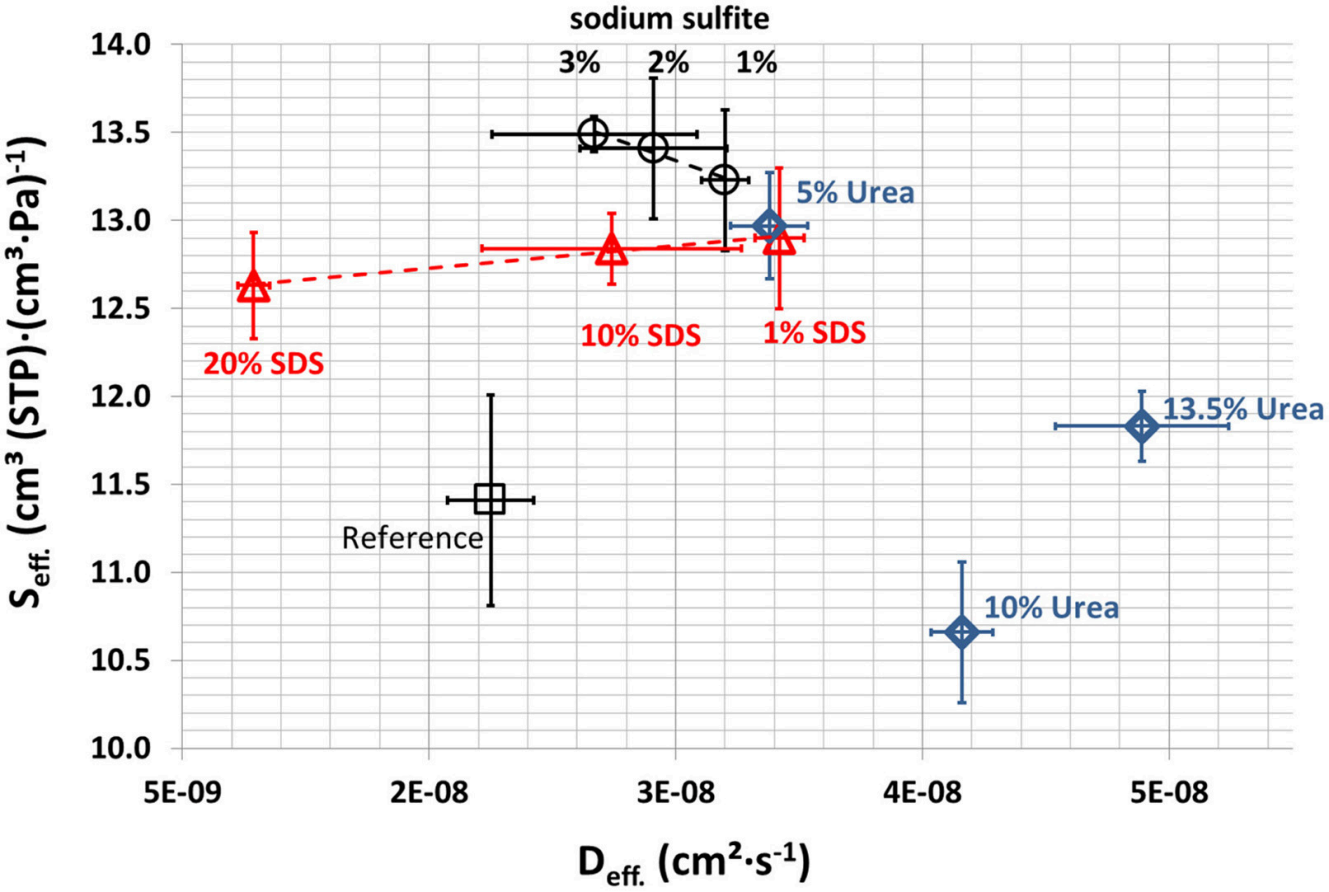
**Effective sorption coefficient and effective diffusion coefficient, of the samples**.

The results from dry film water vapor sorption over time were used to calculate the effective diffusion and the effective solubility coefficients. The results are shown in Figure 10. The effective solubility coefficient and the effective diffusion coefficient increased on adding Na_2_SO_3_, SDS, and urea. A possible explanation is the disturbance of the network, which increases the reaction sites and binding points for water vapor and frees inter-polymer space for diffusion. However, further active binding sites other than hydrogen bonds must be involved because SDS reduces hydrogen bonds. Only the films containing Na_2_SO_3_ showed slightly increased effective solubility coefficients. This might be explained by the fact that the films with Na_2_SO_3_ have an increased relevance of hydrogen bonds, which accelerate the adsorption of water molecules. More hydrophobic interactions due to Na_2_SO_3_ could be the reason for the lower effective diffusion coefficient at higher Na_2_SO_3_ concentration. Reduction of the effective solubility coefficient of SDS is due to the higher number of hydrophobic tails of SDS with increasing SDS concentration, which also explains the lower effective diffusion coefficient because fewer polar groups can bind water molecules that cause the swelling that goes along with higher effective diffusions coefficients. The reduction in the effective solubility coefficient by urea can be explained by the formation of hydrogen bonds with whey proteins, blocking these active sites for reaction with water. Such binding would reduce intermolecular crosslinking which would explain the higher effective water vapor diffusion coefficients.

### Comparison of the water vapor transmission rate measurements and water vapor sorption measurements

**Figure 11:**
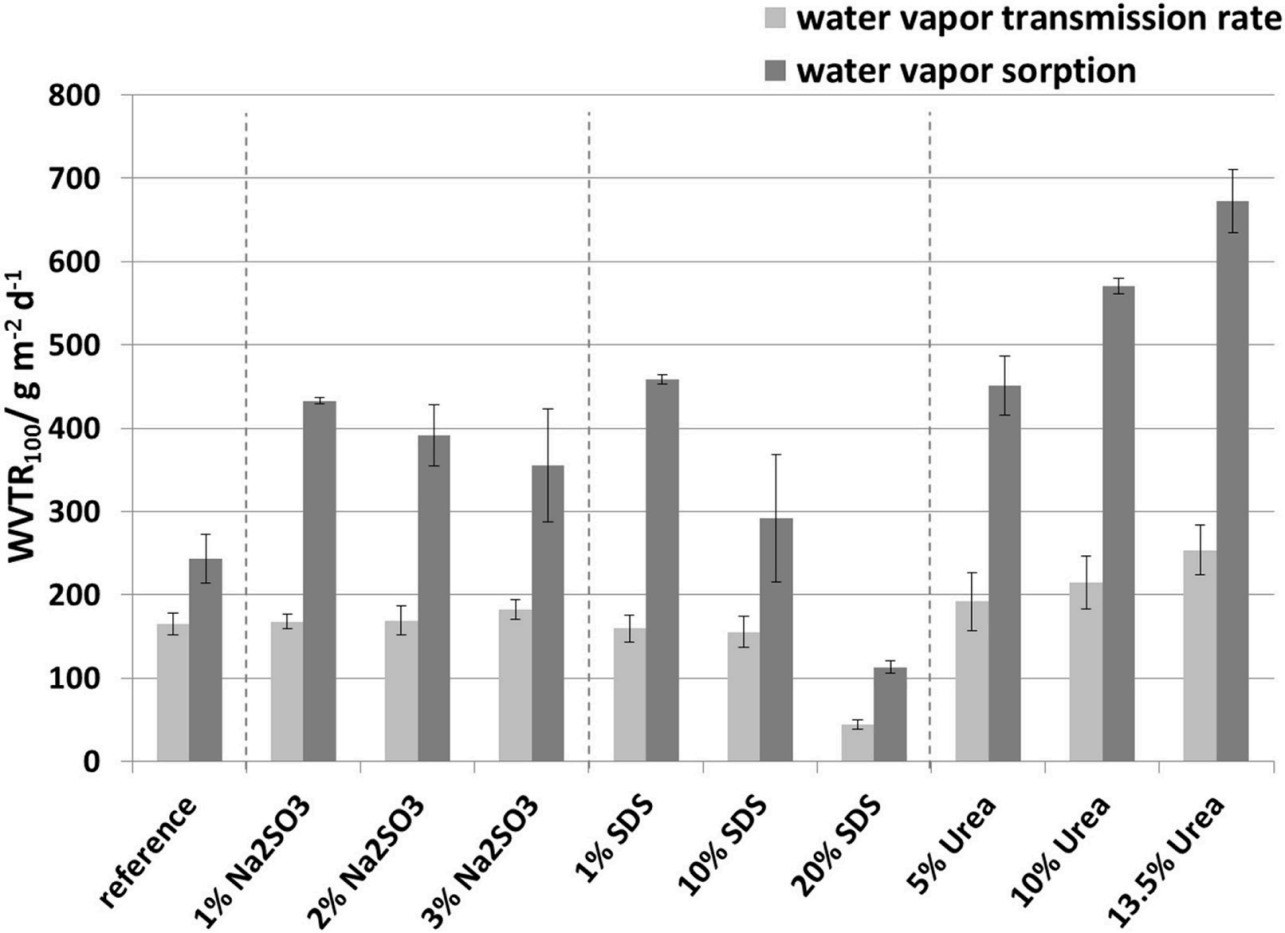
**Comparison of the water vapor transmission rate measurements and water vapor sorption measurements, measured at 50% r.h. and 23°C**.

The water vapor transmission rate (WVTR) from the sorption experiments was calculated from *D*_*eff*_ and *S*_*eff*_ using Equation 3. The results as well as the WVTR determined from the permeation experiments are shown in Figure 11. It is clear that the values calculated from the sorption results were by a factor of 1.5 to 2.9 higher than the values from the water vapor transmission rate measurements. This can be explained by the different methodologies used. The water vapor transmission rate reflects a stationary condition whereas the water vapor sorption experiment reflects an non-stationary condition. Furthermore, the results from the sorption experiments are a simplification, assuming that Fick's laws and the equation derived from it apply. To describe the behavior a more refined model is needed which includes inhomogeneous polymer structures, as is the case for protein films, and takes account of swelling, water molecule cluster formation, non-constant sorption and diffusion coefficients, and relaxation of protein polymer chains. Nevertheless, despite the differences in the water vapor transmission rates both methods showed similar tendencies.

Based on these results, SDS was shown to be the most relevant substance for permeability reduction. At low concentration (1 wt.-%) it reduces the oxygen permeability and at high concentration (20 wt.-%) it increases the oxygen permeability but reduces the water vapor transmission rate by two-thirds. Therefore, further studies should focus on SDS as the active substance. Also, a more suitable mathematic description is needed to more accurately explain the diffusion behavior in the polymer. A possible basis for this could be the random walk theory based on Brown's molecular motion (Brown, 1828; Einstein, 1905, 1906; Philibert, 2006; Helmut Mehrer, 2009; Tejedor, 2012).

## Conclusions

The addition of Na_2_SO_3_, SDS, and urea caused complex interactions and different effects which can in principle counteract each other, such as changed bonding, increased/decreased crosslinking, changes in polarity, and network disturbance by additives. Na_2_SO_3_ did not affect the oxygen permeability and water vapor transmission rate even though the importance of hydrophobic interactions and hydrogen bonds increased and the disulfide bonds decreased slightly. Films with urea showed no change in the intermolecular interactions but the oxygen permeability and water vapor transmission rate increased. For SDS no correlation could be found. SDS proved to be the most relevant substance for permeability reduction. At low concentration (1 wt.-%) it reduces the oxygen permeability and at high concentration (20 wt.-%) it increases the oxygen permeability but reduces the water vapor transmission rate by two- thirds. Therefore, further studies should focus on SDS as the active substance. In addition, a more suitable mathematic description is needed to more accurately explain the diffusion behavior in the polymer.

## Author contributions

MS: Edited the manuscript and was in charge of the outline and experimental plan, evaluation and contributed writing the manuscript. TP: Conducted experiments, contributed writing the manuscript. AS and SS: Evaluated results and contributed writing the manuscript.

### Conflict of interest statement

The authors declare that the research was conducted in the absence of any commercial or financial relationships that could be construed as a potential conflict of interest.
